# Coenzyme Q10 supplementation improves adipokine profile in dyslipidemic individuals: a randomized controlled trial

**DOI:** 10.1186/s12986-022-00649-5

**Published:** 2022-03-03

**Authors:** Peiwen Zhang, Ke Chen, Taiping He, Honghui Guo, Xu Chen

**Affiliations:** 1 Department of Nutrition, School of Public Health, Guangdong Medical University, Dongguan, People’s Republic of China; 2 School of Public Health, Dongguan Key Laboratory of Environmental Medicine, Guangdong Medical University, Dongguan, People’s Republic of China; 3 Department of Nutrition, School of Public Health, Sun Yat-Sen University, Guangzhou, People’s Republic of China

**Keywords:** Coenzyme Q10, Adipokine, Dyslipidemia, Clinical trial, Dietary supplement, Mediating effect

## Abstract

**Background:**

In previous study, we found that coenzyme Q10 (CoQ10) improved glucolipid profile in dyslipidemic individuals, but the mechanism is not yet clear. Adipokines have been demonstrated to be vital targets of metabolic diseases. The hypothesis that adipokines mediate the association of CoQ10 on glucolipid metabolism needs to be further studied in human.

**Methods:**

In this randomized, double-blinded, placebo-controlled trial, 101 dyslipidemic individuals were administrated to 120 mg CoQ10 or placebo for 24 weeks. Anthropometric parameters, glucolipid profile, serum total adiponectin, leptin, and resistin were evaluated at baseline, week 12 and week 24.

**Results:**

CoQ10 treatment significantly increased serum adiponectin levels at week 12 (165 [0, 362] ng/mL, *p* < 0.001) and at week 24 (523 [0, 1056] ng/mL, *p* < 0.001]), which was significant different compared with placebo (*p* < 0.001). The increase of adiponectin was negative associated with decrease in index of homeostasis model assessment of insulin resistance (HOMA-IR, r = − 0.465, *p* = 0.001), triglyceride (TG, r = − 0.297, *p* = 0.047), and low-density lipoprotein cholesterol (LDL-c, r = − 0.440, *p* = 0.002) at week 24 only in CoQ10-treated group. Resistin was reduced by CoQ10 only at week 24 (− 1.19 [− 4.35, 0.00] ng/mL, *p* < 0.001), which was significant different compared with placebo (*p* < 0.001). Reduction of resistin was positively correlated with the change in HOMA-IR (r = 0.343, *p* = 0.021) and TG (r = 0.323, *p* = 0.030) at week 24 in CoQ10-treated group but not placebo group. Leptin was not influenced by CoQ10 treatment. Mediation analysis indicated that the improvement of HOMA-IR, TG and LDL-c by CoQ10 was mediated by adiponectin but not resistin.

**Conclusions:**

Our study shows that CoQ10 ameliorates glucolipid profile and adipokines dysfunction in dyslipidemic patients in 24 weeks’ intervention. The beneficial effect of CoQ10 on glucolipid profile was mediated by adiponectin. Trial registration: ClinicalTrials.gov, NCT02407548. Registered on April 3, 2015, https://clinicaltrials.gov/ct2/show/NCT02407548.

**Supplementary Information:**

The online version contains supplementary material available at 10.1186/s12986-022-00649-5.

## Introduction

Dyslipidemia is a risk factor of cardiovascular disease and vital component of metabolic syndrome. According to national data from 2013–2014, among Chinese adults aged 18 years or older, 28.5%, 26.3 and 25.8% had increased total cholesterol (TC), LDL-c and TG, respectively. And 20.4% had decreased high-density lipoprotein cholesterol (HDL-c) [1]. In developed country such as United States, Japan, and Korea, the incident rate of dyslipidemia is even higher [2–4]. Though the first-line medicine to treat dyslipidemia are statins, which efficiently decrease LDL-c and TC level and risk of atherosclerotic cardiovascular disease by inhibiting cholesterol synthesis. However, the increased risk of developing new-onset diabetes with statin-treatment raised concern of patients with dyslipidemia [5], especially those with borderline hyperlipidemia [6]. More safe and effective measures to improve dyslipidemia and concomitant metabolic disorders deserve investigation.

Accumulating evidences suggest that adipose tissue is an active endocrine organ. By secreting adipokines, such as adiponectin, leptin and resistin, it participates in the crosstalk of organs in various metabolic processes associated with dyslipidemia and hyperglycemia [7]. Adipokines can regulate the inflammation pathways that mediate the lipid metabolism [8]. Some adipokines such as adiponectin can modulate transcription factor such as peroxisome proliferator- activated receptors (PPARs) that regulate gene expression involved in lipid metabolism in multiple tissues [9]. Therefore, Adipokines have also became a pivotal target for treatment of obesity, dyslipidemia and hyperglycemia.

Coenzyme Q10 (CoQ10) is a lipophilic antioxidant, abundant in mammalian organs, such as heart, liver, and kidneys. It forms a crucial part of the electron transport chain in mitochondria which is essential for the production of ATP [10]. CoQ10 levels in the tissues and serum significantly decrease during aging [11] and many other pathological processes, such as myocardial disease [12], degenerative disease [13] and diabetes [14]. The effects of supplementation with CoQ10 in various diseases have been studied, but the results are inconsistent [15, 16]. In previous study, we found that CoQ10 can improve serum lipid profile and insulin resistance in dyslipidemic individuals, but the mechanism was not clear [17]. Is it possible that adipokines would mediate the effect of CoQ10 and improve glucolipid metabolism? Therefore, we measured and conducted further analysis of serum adipokines levels on the basis of our previous study, to clarify the effects of CoQ10 on adipokines and dyslipidemia in human.

## Methods

### Participants

This study used data from the previous randomized, double-blinded, placebo-controlled trial which examined the effects of CoQ10 supplementation on lipid and glycemic profile in dyslipidemic individuals, and a detailed protocol was published [17]. Briefly, 101 individuals were recruited from community health service centers in Guangdong province, China. Participants were included if they were aged from 18 to 70 years and 2 or more of the following serum lipid parameters were abnormal: Serum fasting TC ≥ 5.20 mmol/L (200 mg/dL), fasting TG ≥ 1.70 mmol/L (150 mg/dL), fasting LDL-c ≥ 3.12 mmol/L (120 mg/dL), and fasting HDL-c ≤ 0.91 mmol/L (35 mg/dL). The exclusion criteria included serum fasting TC ≥ 8.0 mmol/L (309 mg/dL); fasting TG ≥ 4.5 mmol/L (395 mg/dL); history of cardiovascular diseases or atherosclerosis; hyperthyroidism or hypothyroidism; cancer; liver or renal dysfunction; consumption of any medicine or dietary supplement that influences lipid and glucose metabolism, inflammation, and oxidative stress.

### Ethics

All protocols in the present study conformed to Helsinki’s Declaration and approved by ethics committee of Sun Yat-Sen University. All subjects in this study were provided written informed consent prior to study entry. This trial had been registered at clinicaltrials.gov as NCT02407548.

### Randomization and intervention

As previously described [17], eligible subjects were recruited and randomized to consume softgels of identical appearance with placebo or 120 mg CoQ10 (4 softgels per day, each contain 30 mg CoQ10, BYHealth Co Ltd, China) for 24 weeks. The softgels were identified by codes printed on the packaging bottles. Participants, investigators, and data analysts were blinded from the grouping information. Randomization was performed by an independent researcher using computer- generated random sequence that matching sex and age in blocks of 4. Participants were requested to maintain their usual diet and exercise habits and visit the study center every 4 weeks. Compliance was assessed by counting the empty pill containers returned by participants at each visit.

### Data collection

Detailed method of data collection have been described in previous published article [17]. Briefly, at baseline, venous blood was collected in the morning after the subjects had fasted for 10–12 h. Then, a structured questionnaire was performed by trained research staffs via face-to-face interview. Information about socio- demographic data, medical history, uses of medications, dietary habits, and physical activities were collected. Blood samples and information collection were repeated at 12 weeks and 24 weeks after intervention.

### Biochemical analyses

After fasting for 10–12 h, blood samples of the subjects were obtained in the morning at the beginning, 12th week, and 24th week of the trial. The blood samples were centrifuged at 3000 × g for 15 min before being separated serum and stored at − 80 °C until used. Biochemical parameters including concentrations of TC, TG, HDL-c, LDL-c, apolipoprotein A-1(ApoA-I), apolipoprotein B (ApoB), blood glucose and insulin were measured with an automatic biochemical analyzer (Roche Group, Switzerland). HOMA-IR index was used to evaluate insulin resistance and calculated as (fasting insulin[mU/L] × fasting blood glucose [mmol/L])/22.5 [18].

Fasting serum total adiponectin was measured using commercial ELISA kits (R&D Systems DRP300, USA). The kit can measure total (low, middle, and high molecular weight) human adiponectin in serum. The average intra and inter-assay coefficients of variation for adiponectin were 5.3% and 6.1%, respectively. Fasting serum leptin was measured by Human Leptin Quantikine ELISA Kit (R&D Systems DLP00, USA). The average intra and inter-assay coefficients of variation for leptin were 3.3% and 8.1%, respectively. Fasting serum resistin was measured with reagents of Human Resistin Quantikine ELISA Kit (R&D Systems DRSN00, USA). The average intra and inter-assay coefficients of variation for resistin were 5.5% and 6.2%, respectively. All ELISA experiments were conducted according to the manufacturer's instructions.

### Statistical analysis

The sample size estimation was based on the primary outcome of TG, TC, LDL-c, and HDL-c as reported in the main paper [17]. Briefly, a sample size of 48 per arm was required to detect a 0.3 mmol/L (26.5 mg/dL) decrease in TG between groups at a type I error of 0.05 (two-tailed) and a type II error of 0.20 (power = 80%) [19]. The sample size estimation of TC, LDL-c, and HDL-c were less than 48. Therefore, at least 48 subjects were needed to include in each group. We conducted an intention-to-treat analysis, which included data from all participants who underwent randomization.

SPSS software (Version19.0, IBM, Inc) was used for statistical analysis. Normality was tested by the Kolmogorov–Smirnov test for continuous variable. Abnormally distributed data were shown as median (with upper and lower quartiles). And differences of such variables between two group were assessed by Mann–Whitney U test. Data with normally distributed were described as mean values (with SD) or mean (with SE) as noted. Differences of such variables between groups were assessed using independent samples t tests. For the categorical variables, the chi-square test and percentage (%) were used. Pearson’s correlation coefficients (r) were calculated to evaluate correlations between the changes in adiponkines and glucolipid metabolic variables. A 2-tailed *p* < 0.05 was considered statistically significant.

**Fig. 1:**
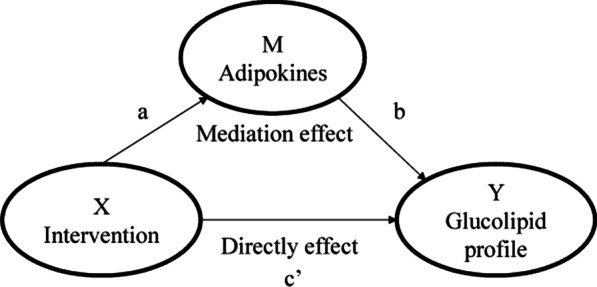
Mediation model for the association between CoQ10 intervention and glucolipid profiles with adipokines as mediators. **a** represents the regression coefficients for the association between intervention grouping and adipokines; **b** represents the regression coefficients for the association between adipokines and glucolipid profiles; a*b equals to the mediation effect of adipokines between intervention grouping and glucolipid profiles; **c’** represents the regression coefficients for the association between intervention grouping and glucolipid profiles, that is the directly effect of them

To test whether the adipokines functioned as mediators between CoQ10 intervention and the improved glucolipid profile, we conducted mediation analysis according to Preacher and Hayes [20], which permits the assessing of multiple indirect effects simultaneously. Mediation analysis implements a series of regression analyses and estimates the total effects (c), direct effect (c’) and the mediation effects (a*b) of a predictor (X) on an outcome of interest (Y) considering the role of one or more variables as mediators (M) (Fig. 1). The 95% confidence interval (CI) of effect were obtained via bootstrapping (5000 bootstrap samples). The 95% CI did not include zero indicating a significant effect. Mediation analyses were performed using Process v2.16.3 by Andrew F. Hayes [20]. All two-sided *p* values < 0.05 were considered statistically significant.

## Results

### General characteristics of the subjects

**Fig. 2:**
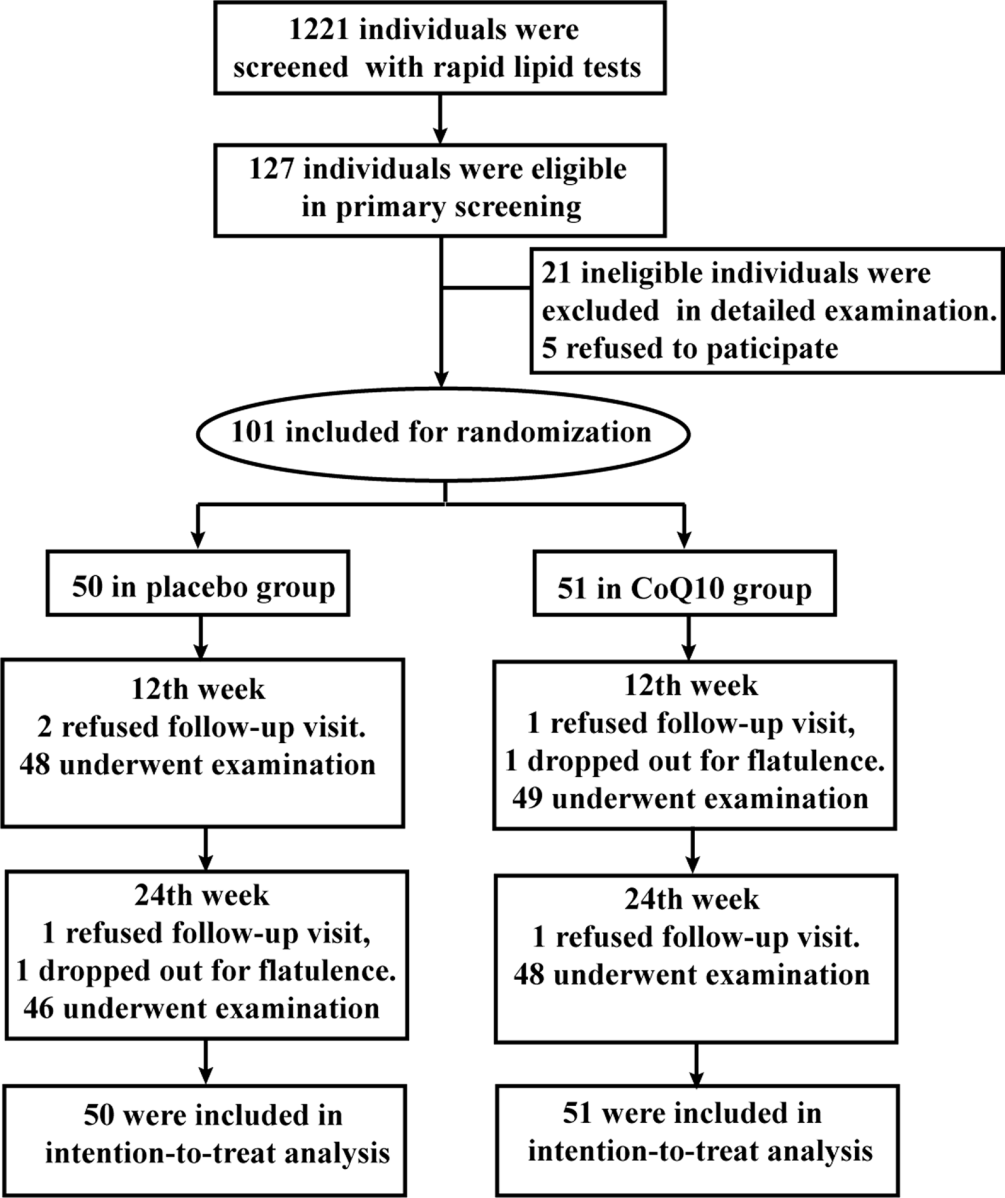
Flow diagram and study design

By using rapid lipid test with CardioChek PA Analyzer (PTS Diagnostics), we screened 127 qualified participants. After detailed examination, 101 were recruited and randomly assigned to either CoQ10 group (n = 51) or placebo groups (n = 50) at baseline. At week 12, two participants lost to follow-up in CoQ10 group for being absent from the schedule visits (n = 1) and flatulence (n = 1). Two participants in placebo group did not attend the schedule visits and withdrew. At week 24, one participants in CoQ10 group cannot meet the schedule visits and withdrew. Two participants withdrew for not coming the schedule visits (n = 1) and flatulence (n = 1) in placebo group. A total of 94 subjects (93.07% of those assigned) completed the study. But all participants who underwent randomization were included in the analysis. The flow chart presented in Fig. 2 shows the allocation and the numbers of dropping out patients for each group.

The mean age of participants included in this study was 50.90 (SD, 9.95 years); 31.7% of them were male. At baseline, mean body weight was 64.26 kg (SD, 13.26 kg), BMI was 25.07 kg/m^2^ (SD, 3.64 kg/m^2^). Intervention of 24 weeks did not change body mass significantly in both group [17]. At baseline, 56.4% of participants were prediabetics (defined as 7.0 > fasting blood glucose ≥ 5.6 mmol/L or 126 > fasting blood glucose ≥ 100.8 mg/dL); 97.0% showed insulin resistance (defined as HOMA-IR index > 1); 64.3% had metabolic syndrome (defined according to National Cholesterol Education Program Adult Treatment Panel III [2005 American Heart Association] revised edition) [21], and the proportion of them were comparable between two groups. At the 24th week, less participants had prediabetes or insulin resistance or metabolic syndrome in CoQ10 group compared with baseline and placebo group (*p* < 0.05, Table 1).

**Table 1:** Number of participants that with metabolic related disorders before and after intervention

Disorders n (%)	Placebo (n = 50)			CoQ10 (n = 51)			*p*_*3*_^c^	*p*_*4*_^d^
	Baseline	24-week	*p*_*1*_^a^	Baseline	24-week	*p*_*2*_^b^		
Prediabetes^e^	32 (64.0)	30 (60.0)	0.680	25 (49.0)	13 (25.5)	0.014	0.125	< 0.001
IR^f^	49 (98.0)	47 (94.0)	0.307	49 (96.1)	40 (78.4)	0.008	0.570	0.024
Mets^g^	30 (60.0)	28 (56.0)	0.685	35 (68.6)	18 (35.3)	0.001	0.365	0.037

^a^*p*_1_ values were calculated by chi-square tests for differences between baseline and 24th week in placebo groups

^b^*p*_2_ values were calculated by chi-square tests for differences between baseline and 24th week in CoQ10 groups

^c^*p*_3_ values were calculated by chi-square tests for differences between two group at baseline

^d^*p*_4_ values were calculated by chi-square tests for differences between two group at 24th week

^e^Defined as 7.0 > fast blood glucose ≥ 5.6 mmol/L or 126 > fast blood glucose ≥ 100.8 mg/dL

^f^IR, insulin resistance, define as HOMA-IR index > 1

^g^Mets, metabolic syndromes, defined according to National Cholesterol Education Program Adult Treatment Panel III (2005 American Heart Association revised edition) definition

### CoQ10 effects on the glucolipid profile

Changes of glucolipid parameters are displayed in Additional file 1: Table S1. Fasting blood glucose, insulin and HOMA-IR did not differ significantly between the intervention groups at baseline or week 12 [17]. At week 24, glucose levels were significantly decreased by the CoQ10 treatment (0.23 mmol/L [SE, 0.09 mmol/L]) compared to the placebo group. There were also significant differences in fasting insulin (2.86 mU/L [SE, 1.29 mU/L]) and the HOMA-IR (0.75, [SE, 0.34]) between the placebo and CoQ10 groups at week 24.

As for lipid profile, there were no significant differences in all markers between the two groups at week 0 and 12 [17]. At week 24, TG (0.33 mmol/L [SE, 0.15 mmol/L]) and LDL-c (0.30 mmol/L [SE, 0.13 mmol/L]) level significantly decreased in CoQ10 group compared to placebo group. ApoA-I/ApoB significantly increased (0.18 mmol/L [SE, 0.04 mmol/L]) in CoQ10 group compared to placebo group.

### CoQ10 effects on serum adipokines

Furthermore, in order to investigate the adipokine changes by CoQ10 intervention, we detected three serum adipokines, which were adiponectin, leptin and resistin at baseline, week 12 and 24. As displayed in Table 2, concentration of three adipokines were not significant different between two intervention groups at baseline. In placebo group, adiponectin slightly but significantly decreased at week 12 (− 78 [− 308, 65] ng/mL, *p* = 0.018), but did not significantly changed at week 24 (0 [− 288, 145] ng/mL, *p* > 0.05) compared to baseline. However, CoQ10 supplementation significantly increased serum adiponectin at week 12 (165 [0, 362] ng/mL, *p* = 0.001) and week 24 (523 [0, 1056] ng/mL, *p* < 0.001) compared to baseline. Change of adiponectin between two groups was significant different at week 12 (*p* < 0.001) and week 24 (*p* < 0.001). Change of resistin concentrations did not differ significantly between the intervention groups at week 12, but significantly decreased in the CoQ10 group (− 1.19 [− 4.35, 0.00] ng/mL) compared to placebo group (0.36 [− 0.37, 1.64] ng/mL) at week 24. Change of leptin levels did not differ significantly between two groups at week 12 or week 24.

**Table 2:** Effect of CoQ10 intervention on adipokines^a^

Adipokines	Placebo group (n = 50)	CoQ10 group (n = 51)	*p*^b^
Adiponectin, ng/mL			
Baseline	5578 (4471, 6939)	5503 (4415, 7561)	0.897
12 week	5648 (4210, 6847)	5668 (4626, 7740)	0.237
24 week	5899 (4308, 6842)	5890 (4848, 8224)	0.154
12-week change^c^	− 78 (− 308, 65)	165 (0, 362)	< 0.001
24-week change^d^	0.00 (− 288, 145)	523 (0, 1056)	< 0.001
Leptin, ng/mL			
Baseline	10.35 (5.98, 16.82)	13.43 (6.09, 20.35)	0.446
12 week	8.37 (6.11, 14.71)	13.98 (6.16, 18.29)	0.156
24 week	8.28 (6.11, 15.21)	12.34 (5.75, 20.19)	0.153
12-week change	0.00 (− 1.79, 0.84)	0.00 (− 0.80, 0.86)	0.728
24-week change	0.00 (− 3.16, 1.77)	0.30 (− 0.10, 3.39)	0.177
Resistin, ng/mL			
Baseline	8.78 (3.79, 14.62)	11.40 (5.67, 14.45)	0.448
12 week	10.55 (4.48, 15.22)	12.14 (6.26, 14.60)	0.526
24 week	10.90 (4.56, 16.31)	6.20 (3.15, 13.15)	0.057
12-week change	0.12 (− 0.45, 1.76)	0.09 (− 0.13, 0.50)	0.868
24-week change	0.36 (− 0.37, 1.64)	− 1.19 (− 4.35, 0.00)	< 0.001

^a^variables are presented as median (with upper and lower quartiles)

^b^*p* value from comparison between two groups using Mann–Whitney U test at baseline, week 12, week 24, 12-week change and 24-week change, respectively

^c^12-week change = adipokine value at week 12—adipokine value at baseline

^d^24-week change = adipokine value at week 24—adipokine value at baseline

### Correlation of adipokines with markers of glucose and lipid metabolism

**Fig. 3:**
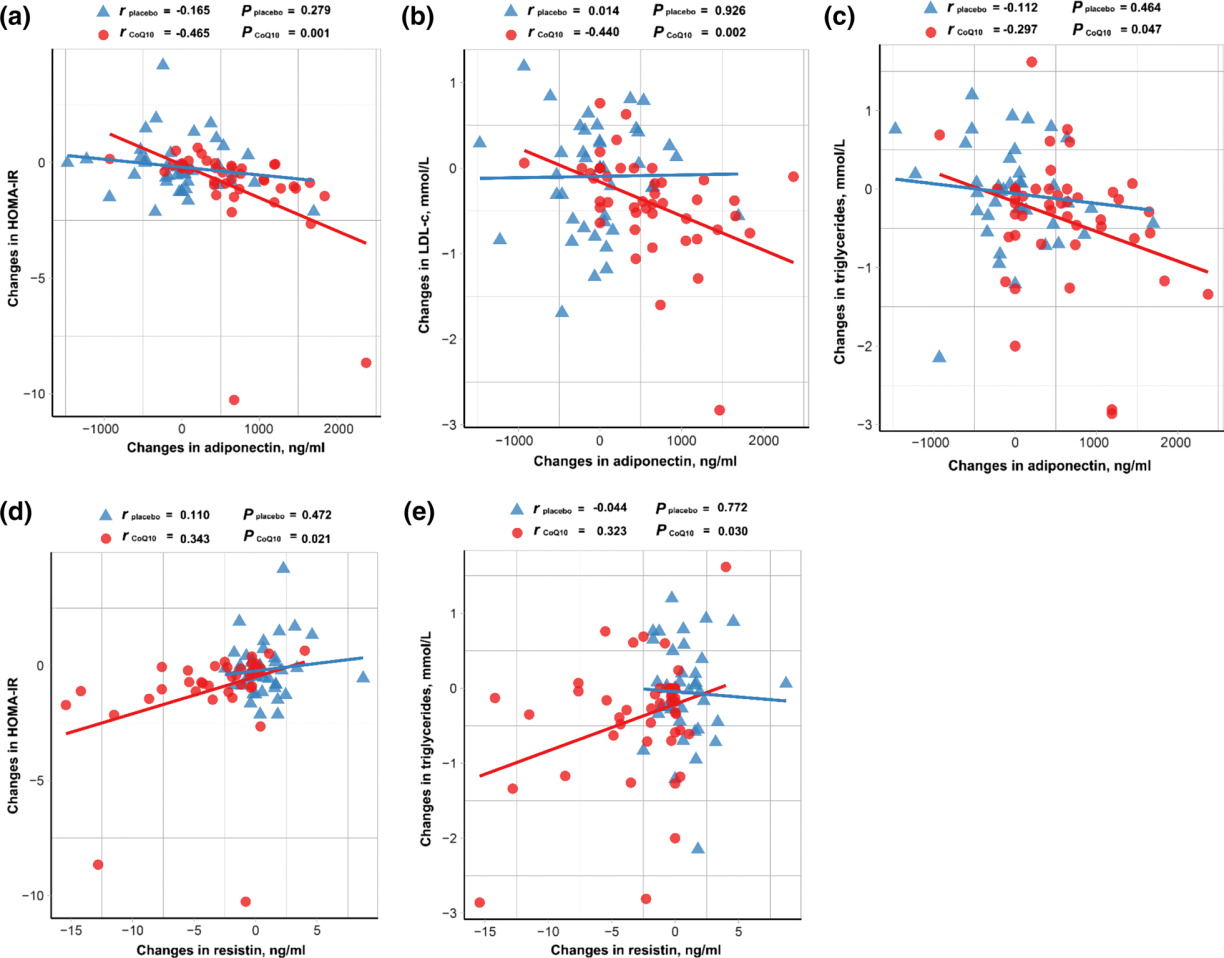
Correlation of adipokines with glucolipid profile. Correlation analysis between the 24-week change in serum adiponectin and HOMA-IR index (**a**), LDL-c (**b**) and TG (**c**) in placebo and CoQ10 group, respectively. Correlation analysis between the 24-week change in serum resistin and HOMA-IR index (**d**) and TG (**e**) in placebo and CoQ10 group, respectively. (n = 50 in placebo and = 51 in CoQ10 group). The data were evaluated by Pearson correlation coefficient (r). HOMA-IR, homeostasis model assessment of insulin resistance; LDL-c, low-density lipoprotein cholesterol; TG, triglyceride

Moreover, we performed a correlation analysis to establish the relationship between adipokine profiles change and glucolipid related markers that had been improved in CoQ10 intervention study. 24-week change of adiponectin was negatively correlated with 24-week change of HOMA-IR (r = − 0.465, *p* = 0.001), TG (r = − 0.297, *p* = 0.047), and LDL-c (r = − 0.440, *p* = 0.002) in CoQ10 group but not placebo group. There was no correlation between change of adiponectin and ApoA-I/ApoB at week 24 in both groups. Change in resistin concentration was positively correlated with the change in HOMA-IR (r = 0.343, *p* = 0.021) and TG (r = 0.323, *p* = 0.030) at week 24 only in CoQ10 group. There was no correlation between change of resistin and ApoA-I/ApoB or LDL-c at week 24 in both groups. Correlations of adipokines with markers of glucose and lipid metabolism are displayed in Fig. 3.

### Mediating effects of adipokines

To further investigate the possible mechanism that adipokines mediated the relationship between CoQ10 and glucolipid metabolism, we performed mediation analysis in significantly correlated adipokines and glucolipid related markers in CoQ10 intervention group. Firstly, we performed simple mediation analysis in the following path way: intervention → adiponectin → HOMA-IR, intervention → resistin → HOMA-IR, intervention → adiponectin → TG, intervention → resistin → TG, intervention → adiponectin → LDL-c. Grouping was set as independent variate X (placebo group = 0, CoQ10 group = 1). 24-week change of HOMA-IR or TG or LDL-c was set as dependent variate Y. Change of adiponectin or resistin between 24 weeks was set as mediator variate M.

As displayed in Table 3, CoQ10 was not directly associated with HOMA-IR or TG or LDL-c (all *p* of direct effect > 0.05). In contrast, the potential effect of CoQ10 on HOMA-IR was mediated by adiponectin (95% CI [− 1.4, − 0.21]) and resistin (95% CI [− 1.19, − 0.08]). However, effect of CoQ10 on TG was mediated by adiponectin (95% CI [− 0.37, − 0.01]) but not resistin (95% CI [− 0.45, 0.04]). Analogously, adiponectin (95% CI [− 0.32, − 0.02]) but not resistin (95% CI [− 0.22, 0.01]) mediated the effect of CoQ10 on LDL-c. As the effect of CoQ10 on HOMA-IR mediated by both adipokines, we further conducted a multiple mediation analysis which included both adiponectin and resistin as mediator variates. Results showed that total mediating effect of both adipokines (95% CI [− 1.60, − 0.29]) and the separate mediating effects of adiponectin (95% CI [− 1.08, − 0.16]) was significant. But the separate mediating effects of resistin (95% CI [− 0.83, 0.05]) was not significant (Table 4).

**Table 3:** The simple mediation effects of adipokines on the association of CoQ10 with glucolipid metabolic markers^a^

Markers	Adiponectin		Resistin	
	Directly effect	Mediation effect	Directly effect	Mediation effect
HOMA-IR^b^	− 0.15 (− 0.86, 0.56)	− 0.60 (− 1.4, − 0.21)	− 0.25 (− 0.98, 0.49)	− 0.50 (− 1.19, − 0.08)
TG^c^	− 0.16 (− 0.50, 0.17)	− 0.17 (− 0.37, − 0.01)	− 0.16 (− 0.49, 0.18)	− 0.17 (− 0.45, 0.04)
LDL-c^d^	− 0.16 (− 0.43, 0.11)	− 0.14 (− 0.32, − 0.02)	− 0.21 (− 0.49, 0.08)	− 0.09 (− 0.22, 0.01)

^a^The CI did not include zero indicates a significant effect

^b^HOMA-IR, homeostasis model assessment of insulin resistance

^c^TG, triglyceride

^d^LDL-c, low-density lipoprotein cholesterol

**Table 4:** The multiple mediation effects of adipokines on the association of CoQ10 with HOMA-IR^a^

Markers	Directly effect	Mediation effect		
		Total	Adiponectin	Resistin
HOMA-IR	0.02 (− 0.72, 0.76)	− 0.76 (− 1.60, − 0.29)	− 0.48 (− 1.08, − 0.16)	− 0.29 (− 0.83, 0.05)

^a^The CI did not include zero indicates a significant effect. HOMA-IR, homeostasis model assessment of insulin resistance

## Discussion

In our current study, supplementation with CoQ10 for 24 weeks not only improved serum levels of glucose, insulin, TG, LDL-c and ApoA-I/ApoB, but also increased serum adiponectin and decreased resistin. In CoQ10 group, change in adiponectin and resistin was correlated with the improvement of glucolipid profile. Moreover, mediating analysis indicated that CoQ10 improve glucolipid metabolism by affecting adiponectin.

As a lipophilic antioxidant, CoQ10 regulated lipid and glucose profile in a series of diseases, such as diabetes [22] and metabolic syndrome [23]. Consistently, our study also concluded that in Chinese dyslipidemia patients, long-term CoQ10 supplementation improved their insulin sensitivity and lipid profile. Though less powerful and cost-effective than clinical medication in lipid lowering and hypoglycemic therapy, CoQ10 has benefits on multiple risk factors of cardiovascular disease, including lowering blood pressure [17], blood glucose, lipids and HOMA-IR with few side effect. Therefore, CoQ10 is quite a good option for those who have moderate dyslipidemia with multiple metabolic disorders.

Leptin is a reliable marker of percentage of fat mass [24]. Increased circulating leptin was observed in insulin resistance and T2DM [25] and correlated positively with lipids levels [26]. In previous studies, CoQ10 supplementation significantly reduced leptin levels in individuals with non-alcoholic fatty liver disease [27] and type 2 diabetes [28], which were inconsistent with our study. The conflicting results may come from that the baseline serum level of leptin in the present study (median, 11.05 ng/mL) was much lower than previous two studies (median, 26.94 ng/mL in patients with non-alcoholic fatty liver disease and 23.51 ng/mL in patients with type 2 diabetes). Accordingly, participants in our study were much thinner (mean BMI was 25.07 kg/m^2^) than those two RCTs (mean BMI was 28.96 kg/m^2^ in patients with non-alcoholic fatty liver disease and 28.99 kg/m^2^ in patients with type 2 diabetes). Our results also shown that CoQ10 did not cause significant weight loss [17]. Therefore, it was not surprising to observe a less remarkable improvement in leptin in subjects who per se had moderate increased of leptin and BMI. However, we cannot totally rule out the possibility that CoQ10 can influence leptin secretion.

Several published RCTs had reported conflicting effect of CoQ10 in adiponectin in non-alcoholic fatty liver disease [27], hypertension [29] and type 2 diabetes [28]. The increase of adiponectin was parallel with the ameliorative effects on lipid peroxidation and glucose control [30]. Results from our present study were consistent with these trials. However, studies conducted in type 2 diabetes [31] and healthy, nonsmoking, sedentary men [32] found that CoQ10 supplementation for 8 weeks showed no improvement in adiponectin. The limited intervention time (less than 12 weeks) and mild illness condition may account for the negative results of adiponectin responded to CoQ10 supplementation.

In this study, we not only found that CoQ10 increased adiponectin, but also found that CoQ10 ameliorated glucolipid profile by mediating adiponectin. Adiponectin was thought as a protective adipokine. Extensive evidence have demonstrated anti-atherosclerotic, anti-diabetic, and anti-inflammatory activities that adiponectin possessed [33]. The gene expression of adiponectin is tightly controlled by a number of factors. PPAR-γ, which is expressed mainly in adipose tissue, is the major positive regulator of adiponectin gene expression. In contrast, inflammation factors such as tumor necrosis factor-alpha (TNF-α) inhibit adiponectin gene expression [34]. Interestingly, CoQ10 intervention can raise the expression of PPAR-γ in peripheral blood mononuclear cells of subjects with polycystic ovary syndrome [35]. CoQ10 can also partially attenuate the effect of TNF-α on PPAR-γ in HL-1 cardiomyocytes [36]. These results further suggested that adiponectin may be an important pathway and target of CoQ10 to improve lipid and glucose metabolic disorders. However, more studies were needed to further confirm them.

In human, resistin is synthesized predominantly by mononuclear cells inside and outside adipose tissues [37, 38]. It can increase the production of the proinflammatory cytokines such as TNF-α and interleukin-6 (IL-6) [39, 40]. As we known, chronic inflammation was involved in the pathogenesis of obesity, type 2 diabetes and atherosclerosis. Therefore, resistin has been suggested as an important modulator and predictor of metabolic diseases [41, 42]. To our knowledge, this is the first study to investigate the effect of CoQ10 on resistin. Supplementation of CoQ10 for 24 weeks reduced serum resistin. Though change in resistin concentration was positive correlated with the change in HOMA-IR and TG in CoQ10 group, mediating analysis showed that resistin did not involve in the regulation mechanism of CoQ10 on these two parameters when considering adiponectin, which indicated that adiponectin is a more important mediator in regulating glucose and lipid. Another possible reason is that the reduction of resistin was accompanied by the improved of glucose and lipid. As resistin has been suggested as a marker of the severity of myocardium ischemic injury [43], the change of resistin by CoQ10 in dyslipidemic patients indicated a further decreased risk for them to develop atherosclerosis.

There were several limitations in this study. Firstly, we did not adjust the diet CoQ10 content as a cofactor in comparison of the effect between two groups. The CoQ10 content in Chinese food have not yet been well examined. However, according to Christine [44], the average CoQ10 intake in the Danish population was estimated to 3–5 mg/day, which was much less than our supplementation dosage (120 mg/d). Moreover, according to 3-day 24-h dietary record, the intake of energy, protein, total fat and total carbohydrate at baseline and during the 24-week intervention of two group was comparable [17]. Adjusted for 12- or 24-week physical activity and energy intake did not change the beneficial effect of CoQ10 on metabolic variables compared to placebo [17]. This suggested that CoQ10 intake by diet did not significantly affect the results of the intervention. Secondly, serum CoQ10 had not been estimated before and after intervention. But we assessed compliance by counting the empty pill containers and inquiry adverse reaction every 4 weeks. Thirdly, we did not deeply investigate the pathway behind CoQ10 and glucolipid metabolism in sophisticated experiment. But the mediation analysis revealed the important mediating role of adiponectin between CoQ10 and glucolipid metabolism. It provided the direction for further research.

## Conclusions

In conclusion, we report that CoQ10 supplementation increase adiponectin and decrease resistin concentrations in dyslipidemic adults, which is correlated with the HOMA-IR and lipid profiles. Data suggested that the improvement of CoQ10 on glucolipid metabolism in dyslipidemic adults was partly by modulating adiponectin.

## Supplementary Information

**Additional file 1: Table S1.** Effect of CoQ10 intervention on glucolipid profile.

## Data Availability

The datasets used and/or analysed during the current study are available from the corresponding author on reasonable request.

## Abbreviations

ApoA-I: Apolipoprotein A-1
ApoB: Apolipoprotein B
CoQ10: Coenzyme Q10
HDL-c: High-density lipoprotein cholesterol
HOMA-IR: Homeostasis model assessment of insulin resistance
IL-6: Interleukin-6
LDL-c: Low-density lipoprotein cholesterol
PPARs: Peroxisome proliferator-activated receptors
TC: Total cholesterol
TG: Triglyceride
TNF-α: Tumor necrosis factor-alpha

## References

[1] Zhang M, Deng Q, Wang L, Huang Z, Zhou M, Li Y, Zhao Z, Zhang Y, Wang L (2018). Prevalence of dyslipidemia and achievement of low-density lipoprotein cholesterol targets in Chinese adults: a nationally representative survey of 163,641 adults. Int J Cardiol.

[2] Lee YH, Lee SG, Lee MH, Kim JH, Lee BW, Kang ES, Lee HC, Cha BS (2014). Serum cholesterol concentration and prevalence, awareness, treatment, and control of high low-density lipoprotein cholesterol in the Korea National Health and Nutrition Examination Surveys 2008–2010: Beyond the Tip of the Iceberg. J Am Heart Assoc.

[3] Carroll MD, Lacher DA, Sorlie PD, Cleeman JI, Gordon DJ, Wolz M, Grundy SM, Johnson CL (2005). Trends in serum lipids and lipoproteins of adults, 1960–2002. JAMA.

[4] Arai H, Yamamoto A, Matsuzawa Y, Saito Y, Yamada N, Oikawa S, Mabuchi H, Teramoto T, Sasaki J, Nakaya N, Itakura H, Ishikawa Y, Ouchi Y, Horibe H, Shirahashi N, Kita T (2006). Prevalence of metabolic syndrome in the general Japanese population in 2000. J Atheroscler Thromb.

[5] Casula M, Mozzanica F, Scotti L, Tragni E, Pirillo A, Corrao G, Catapano AL (2017). Statin use and risk of new-onset diabetes: a meta-analysis of observational studies. Nutr Metab Cardiovasc Dis.

[6] Preiss D, Seshasai SR, Welsh P, Murphy SA, Ho JE, Waters DD, DeMicco DA, Barter P, Cannon CP, Sabatine MS, Braunwald E, Kastelein JJ, de Lemos JA, Blazing MA, Pedersen TR, Tikkanen MJ, Sattar N, Ray KK (2011). Risk of incident diabetes with intensive-dose compared with moderate-dose statin therapy: a meta-analysis. JAMA.

[7] Unamuno X, Gomez-Ambrosi J, Rodriguez A, Becerril S, Fruhbeck G, Catalan V (2018). Adipokine dysregulation and adipose tissue inflammation in human obesity. Eur J Clin Invest.

[8] Vekic J, Zeljkovic A, Stefanovic A, Jelic-Ivanovic Z, Spasojevic-Kalimanovska V (2019). Obesity and dyslipidemia. Metabolism.

[9] Schindler M, Pendzialek M, Grybel KJ, Seeling T, Gurke J, Fischer B, Navarrete SA (2017). Adiponectin stimulates lipid metabolism via AMPK in rabbit blastocysts. Hum Reprod.

[10] Ayer A, Macdonald P, Stocker R (2015). CoQ(1)(0) function and role in heart failure and ischemic heart disease. Annu Rev Nutr.

[11] Kalen A, Appelkvist EL, Dallner G (1989). Age-related changes in the lipid compositions of rat and human tissues. Lipids.

[12] Folkers K, Vadhanavikit S, Mortensen SA (1985). Biochemical rationale and myocardial tissue data on the effective therapy of cardiomyopathy with coenzyme Q10. Proc Natl Acad Sci U S A.

[13] Shults CW, Haas RH, Passov D, Beal MF (1997). Coenzyme Q10 levels correlate with the activities of complexes I and II/III in mitochondria from parkinsonian and nonparkinsonian subjects. Ann Neurol.

[14] Kishi T, Kishi H, Watanabe T, Folkers K (1976). Bioenergetics in clinical medicine. XI. Studies on coenzyme Q and diabetes mellitus. J Med.

[15] Stojanovic M, Radenkovic M (2017). A meta-analysis of randomized and placebo-controlled clinical trials suggests that coenzyme Q10 at low dose improves glucose and HbA1c levels. Nutr Res.

[16] Suksomboon N, Poolsup N, Juanak N (2015). Effects of coenzyme Q10 supplementation on metabolic profile in diabetes: a systematic review and meta-analysis. J Clin Pharm Ther.

[17] Zhang P, Yang C, Guo H, Wang J, Lin S, Li H, Yang Y, Ling W (2018). Treatment of coenzyme Q10 for 24 weeks improves lipid and glycemic profile in dyslipidemic individuals. J Clin Lipidol.

[18] Anderson RL, Hamman RF, Savage PJ, Saad MF, Laws A, Kades WW, Sands RE, Cefalu W (1995). Exploration of simple insulin sensitivity measures derived from frequently sampled intravenous glucose tolerance (FSIGT) tests. The Insulin Resistance Atherosclerosis Study. Am J Epidemiol.

[19] Chew GT, Watts GF, Davis TM, Stuckey BG, Beilin LJ, Thompson PL, Burke V, Currie PJ (2008). Hemodynamic effects of fenofibrate and coenzyme Q10 in type 2 diabetic subjects with left ventricular diastolic dysfunction. Diabetes Care.

[20] Preacher KJ, Hayes AF (2008). Asymptotic and resampling strategies for assessing and comparing indirect effects in multiple mediator models. Behav Res Methods.

[21] Grundy SM, Cleeman JI, Daniels SR, Donato KA, Eckel RH, Franklin BA, Gordon DJ, Krauss RM, Savage PJ, Smith SJ, Spertus JA, Costa F (2005). Diagnosis and management of the metabolic syndrome: an American Heart Association/National Heart, Lung, and Blood Institute Scientific Statement. Circulation.

[22] Mehrdadi P, Kolahdouz MR, Alipoor E, Eshraghian MR, Esteghamati A, Hosseinzadeh-Attar MJ (2017). The effect of coenzyme Q10 supplementation on circulating levels of novel adipokine adipolin/CTRP12 in overweight and obese patients with type 2 diabetes. Exp Clin Endocrinol Diabetes.

[23] Raygan F, Rezavandi Z, Dadkhah TS, Farrokhian A, Asemi Z (2016). The effects of coenzyme Q10 administration on glucose homeostasis parameters, lipid profiles, biomarkers of inflammation and oxidative stress in patients with metabolic syndrome. Eur J Nutr.

[24] Considine RV, Sinha MK, Heiman ML, Kriauciunas A, Stephens TW, Nyce MR, Ohannesian JP, Marco CC, McKee LJ, Bauer TL, Et A (1996). Serum immunoreactive-leptin concentrations in normal-weight and obese humans. N Engl J Med.

[25] Bidulescu A, Dinh PJ, Sarwary S, Forsyth E, Luetke MC, King DB, Liu J, Davis SK, Correa A (2020). Associations of leptin and adiponectin with incident type 2 diabetes and interactions among African Americans: the Jackson heart study. BMC Endocr Disord.

[26] Ostlund RJ, Yang JW, Klein S, Gingerich R (1996). Relation between plasma leptin concentration and body fat, gender, diet, age, and metabolic covariates. J Clin Endocrinol Metab.

[27] Farsi F, Mohammadshahi M, Alavinejad P, Rezazadeh A, Zarei M, Engali KA (2016). Functions of coenzyme Q10 supplementation on liver enzymes, markers of systemic inflammation, and adipokines in patients affected by nonalcoholic fatty liver disease: a double-blind, placebo-controlled, randomized clinical trial. J Am Coll Nutr.

[28] Gholami M, Zarei P, Sadeghi Sedeh B, Rafiei F, Khosrowbeygi A (2018). Effects of coenzyme Q10 supplementation on serum values of adiponectin, leptin, 8-isoprostane and malondialdehyde in women with type 2 diabetes. Gynecol Endocrinol.

[29] Bagheri NN, Mozaffari-Khosravi H, Najarzadeh A, Salehifar E (2015). The effect of coenzyme Q10 supplementation on pro-inflammatory factors and adiponectin in mildly hypertensive patients: a randomized, double-blind, placebo-controlled trial. Int J Vitam Nutr Res.

[30] Dludla PV, Orlando P, Silvestri S, Marcheggiani F, Cirilli I, Nyambuya TM, Mxinwa V, Mokgalaboni K, Nkambule BB, Johnson R, Mazibuko-Mbeje SE, Muller C, Louw J, Tiano L. Coenzyme Q10 supplementation improves adipokine levels and alleviates inflammation and lipid peroxidation in conditions of metabolic syndrome: a meta-analysis of randomized controlled trials. Int J Mol Sci 2020;21. 10.3390/ijms21093247 PMC7247332 32375340

[31] Moazen M, Mazloom Z, Ahmadi A, Dabbaghmanesh MH, Roosta S (2015). Effect of coenzyme Q10 on glycaemic control, oxidative stress and adiponectin in type 2 diabetes. J Pak Med Assoc.

[32] Gokbel H, Gergerlioglu HS, Okudan N, Gul I, Buyukbas S, Belviranli M (2010). Effects of coenzyme Q10 supplementation on plasma adiponectin, interleukin-6, and tumor necrosis factor-alpha levels in men. J Med Food.

[33] Maeda N, Funahashi T, Matsuzawa Y, Shimomura I (2020). Adiponectin, a unique adipocyte-derived factor beyond hormones. Atherosclerosis.

[34] Fang H, Judd RL (2018). Adiponectin regulation and function. Compr Physiol.

[35] Rahmani E, Jamilian M, Samimi M, Zarezade MM, Aghadavod E, Akbari E, Tamtaji OR, Asemi Z (2018). The effects of coenzyme Q10 supplementation on gene expression related to insulin, lipid and inflammation in patients with polycystic ovary syndrome. Gynecol Endocrinol.

[36] Lee TI, Kao YH, Chen YC, Chen YJ (2009). Proinflammatory cytokine and ligands modulate cardiac peroxisome proliferator-activated receptors. Eur J Clin Invest.

[37] Patel L, Buckels AC, Kinghorn IJ, Murdock PR, Holbrook JD, Plumpton C, Macphee CH, Smith SA (2003). Resistin is expressed in human macrophages and directly regulated by PPAR gamma activators. Biochem Biophys Res Commun.

[38] Bo S, Gambino R, Pagani A, Guidi S, Gentile L, Cassader M, Pagano GF (2005). Relationships between human serum resistin, inflammatory markers and insulin resistance. Int J Obes (Lond).

[39] Bokarewa M, Nagaev I, Dahlberg L, Smith U, Tarkowski A (2005). Resistin, an adipokine with potent proinflammatory properties. J Immunol.

[40] Nagaev I, Bokarewa M, Tarkowski A, Smith U (2006). Human resistin is a systemic immune-derived proinflammatory cytokine targeting both leukocytes and adipocytes. PLoS ONE.

[41] Reilly MP, Lehrke M, Wolfe ML, Rohatgi A, Lazar MA, Rader DJ (2005). Resistin is an inflammatory marker of atherosclerosis in humans. Circulation.

[42] Steppan CM, Bailey ST, Bhat S, Brown EJ, Banerjee RR, Wright CM, Patel HR, Ahima RS, Lazar MA (2001). The hormone resistin links obesity to diabetes. Nature.

[43] Chu S, Ding W, Li K, Pang Y, Tang C (2008). Plasma resistin associated with myocardium injury in patients with acute coronary syndrome. Circ J.

[44] Weber C, Bysted A, Holmer G (1997). Coenzyme Q10 in the diet–daily intake and relative bioavailability. Mol Aspects Med.

