# Supplementary material for: Coenzyme Q10 supplementation improves adipokine profile in dyslipidemic individuals: a randomized controlled trial
Source: Nutr Metab (Lond). 2022 Mar 3;19:13. doi: 10.1186/s12986-022-00649-5 (PMC8896379; doi:10.1186/s12986-022-00649-5)
Supplement: Supplementary file 1 — Additional file 1: Table S1. Effect of CoQ10 intervention on glucolipid profile. [file 12986_2022_649_MOESM1_ESM.doc]

**Supplemental Table 1. Effect of CoQ10 intervention on** **glucolipid profile**

| Characteristic | Change in CoQ10 – Change in placebo Mean (SE) | | | *p_1_^a^* | *p_2_^b^* |
| --- | --- | --- | --- | --- | --- |
|  | At 12 week |  | At 24 week |  |  |
| Glucose, mmol/L | -0.08 (0.11) |  | -0.23 (0.09) | 0.496 | 0.011 |
| Insulin, mU/L | -1.33 (0.73) |  | -2.86 (1.29) | 0.071 | 0.030 |
| HOMA-IR | -0.38 (0.23) |  | -0.75 (0.34) | 0.099 | 0.031 |
| TG ^c^, mmol/L | -0.07 (0.14) |  | -0.33(0.15) | 0.626 | 0.032 |
| Cholesterol, mmol/L | -0.19 (0.17) |  | -0.12 (0.14) | 0.271 | 0.406 |
| HDL-c ^d^, mmol/L | -0.03 (0.06) |  | 0.02 (0.04) | 0.597 | 0.614 |
| LDL-c ^e^, mmol/L | -0.17 (0.13) |  | -0.30 (0.13) | 0.181 | 0.020 |
| ApoA-I ^f^, g/L | 0.02 (0.05) |  | 0.20 (0.04) | 0.620 | <0.001 |
| ApoB ^g^, g/L | -0.02 (0.04) |  | -0.02 (0.03) | 0.558 | 0.464 |
| ApoA-I / ApoB | 0.05 (0.06) |  | 0.18 (0.04) | 0.428 | <0.001 |

*^a^ p* value from comparison between two groups using independent samples t tests at week 12.

*^b^ p* value from comparison between two groups using independent samples t tests at week 24.

^c^ TG is short for triglyceride

^d^ HDL-c is short for high-density lipoprotein cholesterol

^e^ LDL-c is short for low-density lipoprotein cholesterol

^f^ ApoA-I is short for apolipoprotein A-1

^g^ ApoB is short for apolipoprotein B
